# The safety and effectiveness of Santulli enterostomy in neonatal intestinal conditions

**DOI:** 10.3389/fped.2022.1077346

**Published:** 2023-01-04

**Authors:** Ming Yue, Heying Yang, Mingxia Cui, Yuhang Yuan, Ning Zhang, Xiangyu Zhang, Yan Li

**Affiliations:** Pediatric Surgery Department, The First Affiliated Hospital of Zhengzhou University, Zhengzhou, China

**Keywords:** Santulli enterostomy, neoborn, emergency surgery, intestinal atresia, necrotizing enterocolitis, anastomosis

## Abstract

**Background:**

As an end stoma, Santulli enterostomy provides early restoration of intestinal continuity without formal laparotomy. Short amputation of the common limb enables closure on a side to restore anatomic continuity without sacrificing valuable intestine; additionally, the procedure is simple and safe. Most newborns who require enterostomy might benefit from Santulli enterostomy; however, several pediatric surgeons lack information regarding this procedure. Therefore, we have reviewed our experience about Santulli enterostomy and explore the advantages and indications in neonatal intestinal conditions.

**Methods:**

The clinical data of 76 neonates who underwent enterostomywere obtained. The patients were divided into two groups: the Santulli group with 33 cases who underwent Santulli enterostomy, and the control group with 43 cases who underwent double- or single-lumen ostomy. The general data of the two groups were analyzed, and the perioperative/postoperative complications, clinical data and the long-term outcomes were compared.

**Results:**

There was no difference in the demographic informations, the level of enterostomy, the rate of high-sight stoma, the operative time and bleeding of enterostomy between the two groups. Compared to the control group, the operative time of ostomy closure was less in the Santulli group (53.00 vs. 152.47, *P* < 0.001). The duration of parenteral nutrition (27.45 vs. 44.56, *P* = 0.010), the mean interval of initial enterostomy to stomal closure (131.21 vs. 216.42, *P* < 0.001), and length of stay (46.00 vs. 67.60, *P* = 0.007) were shorter, while the incidence of postoperative complications and hospitalization costs (11.21 vs. 15.49, *P* = 0.006) were lower. The Santulli procedure can reduce the morbidity of high output ostomy (2 vs. 10, *P* = 0.042) and short bowel syndrome (3 vs. 132, *P* = 0.025), shorten the discrepancy of diameter between the proximal and distal segments, maximize the available intestine, and monitor the movement of the distal bowel. The length of incision was shorter, and the catch-up growth was significantly faster in the Santulli group.

**Conclusion:**

Santulli enterostomy is a superior procedure in the treatment of neonatal intestinal conditions, in terms of fewer complications, faster catch-up growth, shorter hospitalization time and treatment duration. It should be the procedure of choice in several newborns with intestinal conditions that require ostomy.

## Introduction

Intestinal stomas are frequently used in surgical procedures for the temporary treatment of congenital intestinal malformations and postnatal intestinal injuries (1, 2). The varieties of stoma configurations all have specific indications and potential complications. Santulli enterostomy was first described by Santulli in 1961 for the treatment of necrotizing enterocolitis (NEC) in children. This procedure allowed immediate and continued decompression of the obstructed bowel, and anastomotic function was attained by intermittent occlusion of the enterostomy beginning on the fifth postoperative day. Santulli reported an increase in survival from 40% to 80% in the surgical management of intestinal atresia (3). To the best of our knowledge, few reports about Santulli enterostomy have been published since the original series (4–7). Moreover, it has not been widely used since several surgeons have inadequate information about it.

At our institution, we have observed adverse events in different surgical options for neonatal intestinal obstruction and found that Santulli enterostomy is superior to other procedures. With this accumulated experience, this technique has been attempted more frequently for various indications. This study aimed to evaluate and compare the clinical efficacy between Santulli enterostomy and double-/single-lumen ostomy in various emergency surgical conditions in newborns who required stoma. Herein, we report our experience to help more neonatal surgeons to understand and perform this technique. This report will enable newborns with intestinal conditions, which require stoma, to benefit from this procedure in the future.

## Materials and methods

### Study populations

This retrospective analysis study received ethical approval from the Ethics Committee of First Affiliated Hospital of Zhengzhou University (2019-KY-351). All patients had been diagnosed and treated for their neonatal intestinal obstruction that necessitated stoma by a homogeneous group of anesthetists, neonatologists, and pediatric surgeons at the First Affiliated Hospital of Zhengzhou University, in the years 2015–2022.

The inclusion criteria were as follows: ① neonates with intestinal conditions, diagnosed with necrotizing enterocolitis, intestinal atresia, meconium ileus, midgut volvulus, or HD; ② primary anastomosis was presumed to be unwise for a variety of reasons; ③ neonates who underwent enterostomy.

The exclusion criteria were as follows: neonates who: ① were older than 28 days; ② had a combination of other congenital anomalies; ③ underwent operation before admission; ④ underwent colostomy; ⑤ were lost to follow-up; and ⑥ had inadequate data.

A total of 76 patients with neonatal intestinal conditions, who had been diagnosed by a pediatric surgeon based on clinical and paraclinical findings and met the inclusion/exclusion criteria, were included. Based on the surgical description, the cases were divided into two surgical groups: 33 cases in the Santulli group and 43 cases in the control group.

The study evaluated the demographic information of the study participants. Additionally, the surgical outcomes of the neonates who underwent Santulli ostomy were compared to cases submitted to double-/single-lumen ostomy. In the Santulli group, the proximal loop of the divided bowel was brought out as an ostomy and the distal end was re-anastomosed to the proximal bowel from 5 cm proximal to the ostomy site. In the control group, 13 cases were managed by double- lumen ostomy, 30 cases were managed single-lumen ostomy during the initial surgery.

### Variables

All the medical charts were reviewed and clinical data were collected. The demographic information included age, gender, diagnosis, nutritional status, body weight at different periods of time, and the *Z* quality score.

The perioperative items included the operative time, the level of enterostomies (the distance between stomal vent and duodenojejunal junction), rate of high-sight stoma (the distance between stomal vent and duodenojejunal junction <60 cm), duration of TPN, interval between the enterostomy and closure, diameter ratio of the proximal end to the distal end, and the length of stay (LOS).

The day of starting oral or nasal feeding, the length of hospital stay after stoma close and postoperative complications, including skin problems (rash, excoriation, and frank skin breakdown), stoma-related problems (prolapse, retraction, and stricture), anastomotic leaks, high output ostomy, adhesive intestinal obstruction, short bowel syndrome, nutrition station, PN-associated complications (nutrition-associated cholestasis, catheter-associated sepsis) and hospitalization indexes were evaluated in both groups. We had tested serum electrolyte and nutritional indexes at the 1st, 4th, 7th days, the 1st, 2nd, 3rd, 6th, 9th, 12th months after surgery, additionally, measured them 1–5 times per week on the periods of PN usage.

All patients received regular follow-up at 1, 2, 3, 6, 12, and 24 months in the first 2 years after discharge and once a year since. The median follow-up period was 36 months (range 12–72 months). adhesive ileus, length of incision, percentiles for weight were recorded, there were no missing data in the institution.

### Statistical analysis

Statistical analysis is performed using R language (v3.5.2). Categorical parameters were analyzed using the Pearson's chi-square test or Fisher's exact test. The Student's two-tailed *t*-test was used for numeric variables. A *P*-value <0.05 was considered as statistically significant.

## Results

### Baseline characteristics/demographics

The two groups were compared with respect to the sex, gestational age, age, *Z* quality score, body weight and diagnosis at stoma (Table 1), there were no significantly difference.

**Table 1:** Demographics.

Parameter	Santulli group (*n* = 33)	Control group (*n* = 43)	*χ*^2^/*t*	*P*
**Gender (*n*, %)**			0.959	0.172
Male	18 (54.55)	30 (69.77)		
Female	15 (45.45)	13 (30.23)		
**GA (w)**	33.49 ± 4.20	34.14 ± 4.32	0.662	0.510
**Age (days)**	7.06 ± 8.81	10.84 ± 12.08	1.575	0.120
**Body weight (kg)**				
At birth	2.36 ± 0.93	2.36 ± 1.15	0.012	0.990
At enterostomy	2.63 ± 1.74	2.75 ± 1.51	0.322	0.322
**Z quality score**				
Stoma	−0.61 ± 1.22	−0.62 ± 1.39	0.038	0.970
Closure	−0.47 ± 1.35	−0.63 ± 1.79	0.443	0.659
**Diagnosis (*n*, %)**			4.109	0.392
NEC	9 (27.27)	17 (39.53)		
Intestinal atresia	11 (33.33)	11 (25.58)		
HD	3 (9.09)	8 (18.60)		
Midgut volvulus	2 (6.06)	1 (2.33)		
Meconium ileus	8 (24.24)	6 (13.95)		

GA, gestational age; Z quality score (weight-for-age) = (weight-mean)/SD (8–10); NEC, necrotizing enterocolitis; HD, hirschsprung's disease.

### Perioperative course

In the two groups, there was no difference with respect to the level of enterostomies, rate of high-sight stoma, bleeding and the operative time of enterostomy. Meanwhile, bleeding of the stoma closure was lesser and the operative time of stoma closure was shorter in the Santulli group.

As compared with the control group, the interval between enterostomy and closure, the duration of parenteral nutrition, and the length of stay in the Santulli group were significantly shorter (Table 2).

**Table 2:** Variables during perioperative period of two groups.

Variables	**Santulli** group	Control group	*χ*^2^/***t***	*P*
IBEAC/days	131.21 ± 46.43	216.42 ± 123.44	4.159	<0.001
Body weight/kg	7.22 ± 4.51	7.97 ± 2.65	0.855	0.396
stoma sight/cm	82.8 ± 34.60	89.53 ± 30.80	0.871	0.387
high-sight stoma/*n* (%)	11 (33.33)	9 (20.93)	1.481	0.225
**Operative time/min**				
Stoma	136.30 ± 49.21	119.00 ± 43.29	1.627	0.108
Closure	53.00 ± 19.92	152.47 ± 60.08	10.154	<0.001
**Bleeding/ml**				
Stoma	7.70 ± 8.99	11.07 ± 15.17	1.208	0.231
Closure	4.70 ± 3.77	15.93 ± 13.27	5.281	<0.001
PN/days	27.45 ± 16.61	44.56 ± 37.93	2.645	0.010
**DROPETD**				
Stoma	9.77 ± 1.26	9.45 ± 1.81	0.479	0.638
Closure	2.48 ± 0.66	5.91 ± 1.51	6.889	<0.001

Body weight, body weight at stoma close; Stoma sight, the distance between vent of stoma and duodenojejunal junction; PN, parenteral nutrition; BEAC, interval between enterostomy and closure; LOS, length of stay; DROPTDE, diameter ratio of proximal end to distal in intestinal atresia.

**Figure 1:**
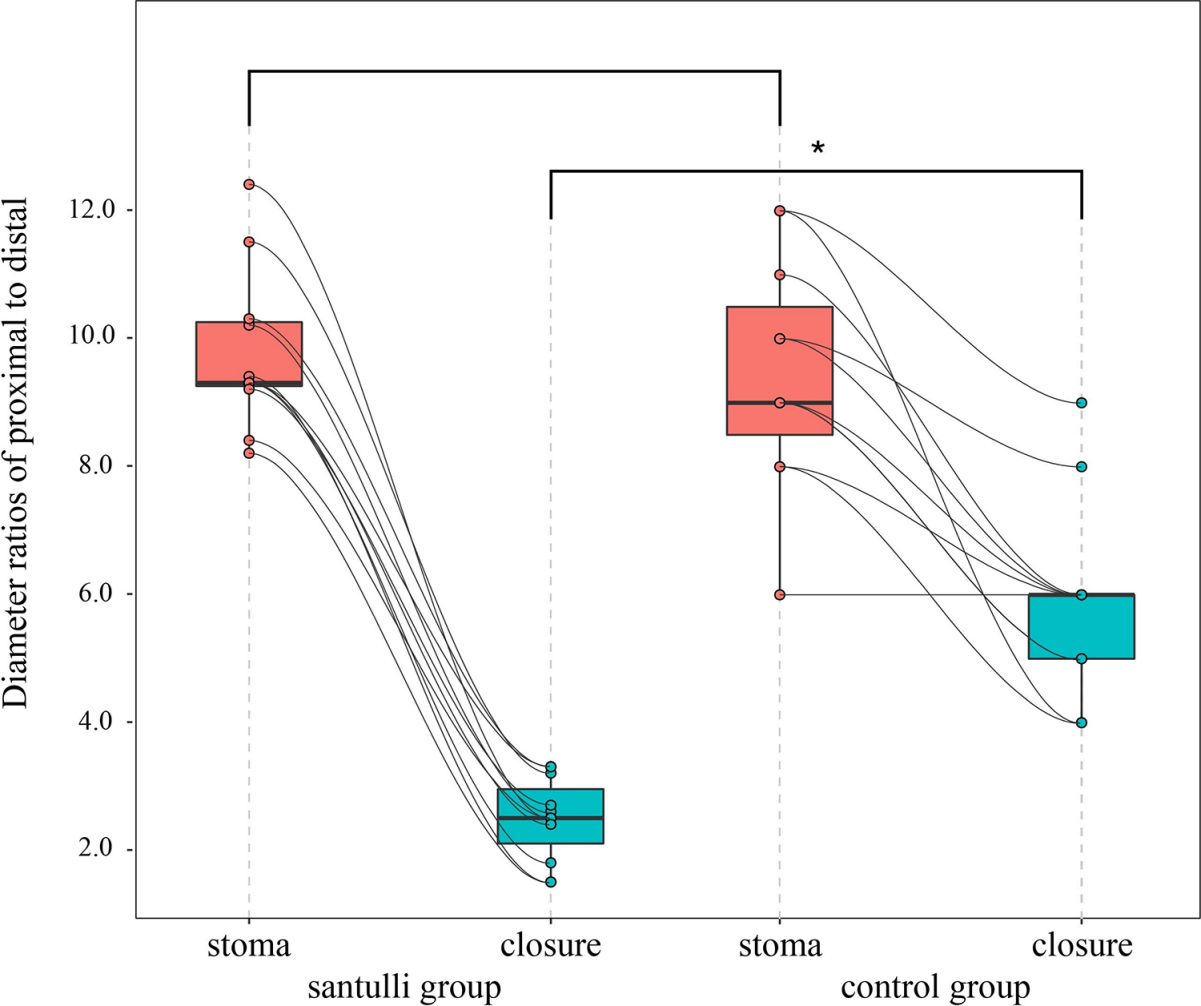
Diameter ratio of proximal end to distal in intestinal atresia at stoma and clousure. *There is a statistical difference.

The diameter ratio of the proximal to distal end in intestinal atresia is similar in two groups at stoma (9.77 vs. 9.45, *P* = 0.638); however, the diameter ratio at closure was smaller (2.48 vs. 5.91, *P* < 0.001) in the Santulli group and the difference was statistically significant (Figure 1).

### Postoperative data

Upon comparison of the two groups, the results showed that both the total complication rate and each complication rate in the Santulli group were lower (Table 3). While there were no difference in the stoma-related problems, intestinal obstruction, anastomotic leak, PN-associated complications, and skin problems. the incidence rate of short bowel syndrome (SBS), high output ostomy, and malnutrition in the Santulli group were lower, and it was statistically significant. Compared to the control group, the day of starting oral or nasal feeding (4.27 ± 1.79 vs. 5.44 ± 2.35, *P* = 0.016) and the length of hospital stay after stoma close (7.64 ± 2.29 vs. 11.58 ± 5.41, *P* < 0. 001) was lesser in the santulli group.

**Table 3:** Postoperative data (*n*, %).

Parameter	Santulli group (*n* = 33)	Control group (*n* = 43)	*χ* ^2^	*P*
First feeding time	4.27 ± 1.79	5.44 ± 2.35	2.460	0.016
Length of stay	7.64 ± 2.29	11.58 ± 5.41	4.307	<0.001
**Complications**				
SBS	3 (9.09)	13 (30.23)	5.021	0.025
^F^Stoma problems	2 (6.06)	6 (11.63)	----	0.454
Malnutrition	5 (15.15)	16 (37.21)	4.543	0.033
^C^Intestinal obstruction	3 (9.09)	7 (16.28)	0.332	0.564
^F^Anastomotic leak	0	4 (9.30)	----	0.128
high-output stoma	2 (6.06)	10 (23.26)	4.152	0.042
^C^PN-associated complications	2 (6.06)	8 (18.60)	1.591	0.207
Skin problems	6 (18.18)	12 (27.91)	0.977	0.323
Total	23 (69.70)	86 (200)	5.418	0.020

First feeding time:the day of starting oral or nasal feeding; Length of stay:length of hospital stay after stoma close skin problems:include rash, excoriation, and frank skin breakdown; stoma problems: prolapse/retraction/stricture; High-output stoma:the stomal output >40 ml/kg·days at 4th week after stoma; PN-associated complications:include Catheter-associated sepsis, nutrition-associated cholestasis; F, Fisher exact probability method. C, *χ*^2^ continuity correction formula.

The potassium, sodium and Magnesium were similar in the comparisons of the serum electrolyte between the two groups (Table 4). hypocalcemia and acidosis were significantly severer in the control group, and all the nutritional indexes, including Retinol-binding protein, Prealbumin and Vitamin B_12_, in the santulli group were significantly better than that of the control group.

**Table 4:** The serum electrolyte and nutritional indexes.

Parameter	Santulli group	Control group	*t*	*P*
Potassium (mmol/L	3.59 ± 0.59	3.55 ± 0.61	1.067	0.286
Sodium (mmol/L)	136.00 ± 5.05	136.43 ± 6.06	1.285	0.200
Calcium (mmol/L)	2.11 ± 0.50	1.96 ± 0.58	4.859	<0.001
Magnesium (mmol/L)	0.62 ± 0.13	0.61 ± 0.15	0.533	0.594
Bicarbonate (mmol/L)	20.84 ± 3.51	20.04 ± 6.52	2.655	0.008
Retinol-binding protein (mg/L)	9.29 ± 3.58	8.42 ± 4.95	3.425	0.001
Prealbumin (mg/L)	130.85 ± 51.60	96.12 ± 58.98	10.490	<0.001
Vitamin B_12_ (pmol/L)	234.10 ± 216.68	287.20 ± 199.64	4.243	<0.001

### Hospitalization indexes

**Figure 2:**
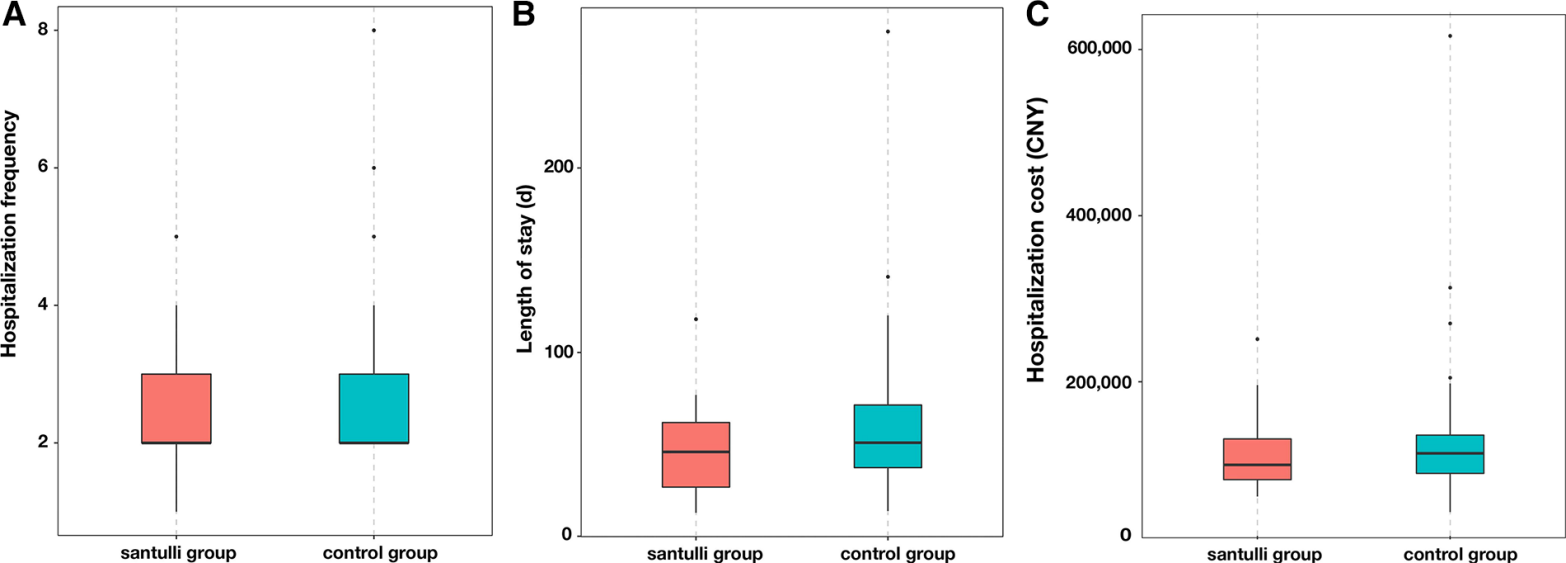
Box and whisker plots of the hospitalization indexes in two groups: (**A**) hospitalization frequency, (**B**) length of stay, and (**C**) hospitalization cost.

The hospitalization frequency was fewer (2.33 ± 0.85 vs. 2.79 ± 1.32, *P* = 0.072) in the Santulli group than the control group (Figure 2A), although there was no significant difference. Both the length of stay (46.00 ± 23.29 days in the Santulli group vs. 67.60 ± 42.32 days in the control group, *P* = 0.007, Figure 2B) and hospitalization cost (112,100 ± 43,100 CNY in the Santulli group vs. 154,900 ± 85,100 CNY in the control group, *P* = 0.006, Figure 2C) were fewer in the Santulli group.

### Long-term outcomes

**Figure 3:**
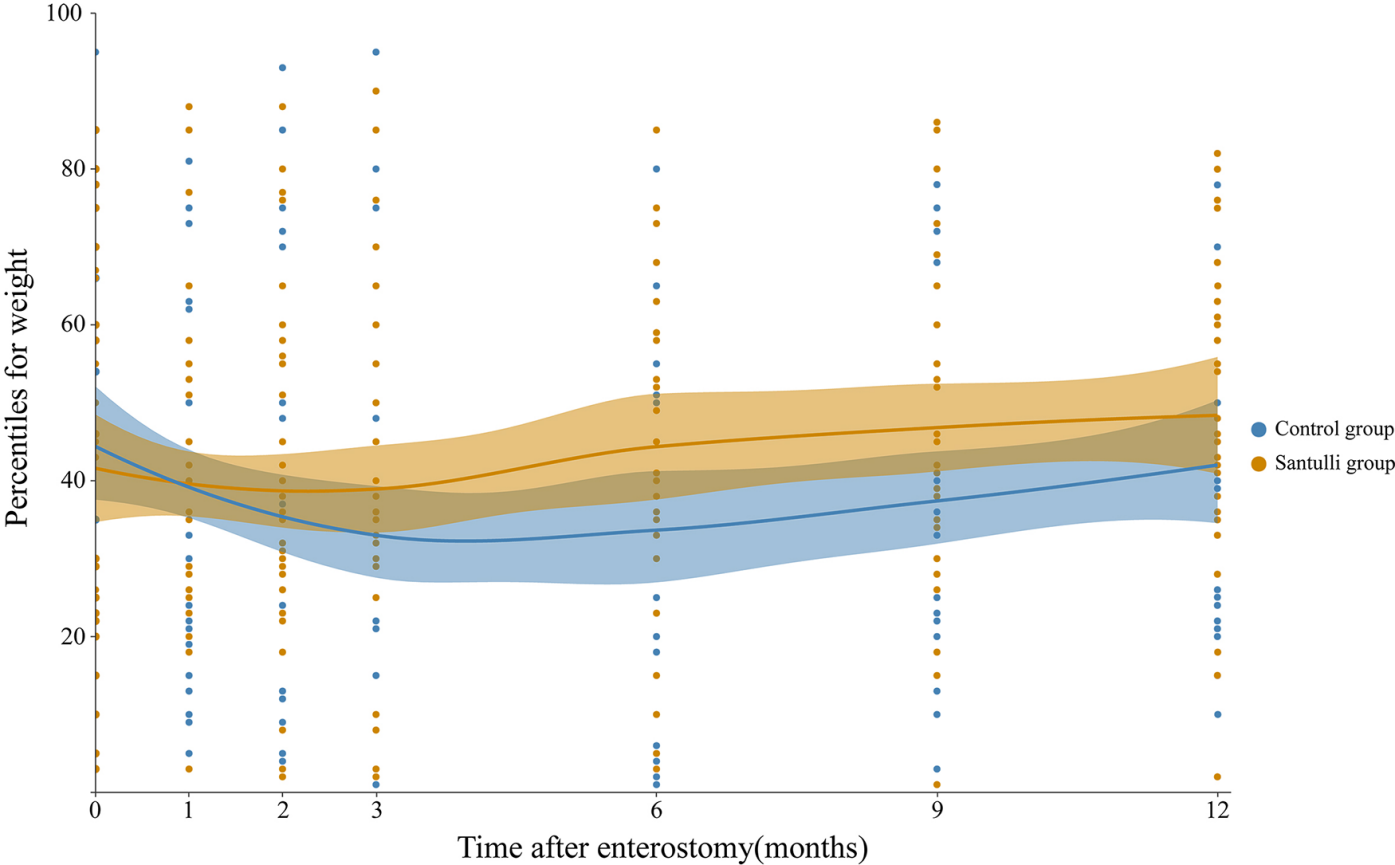
The percentiles for weight after surgery during different periods of time.

There were no difference with respect to adhesive ileus, and percentiles for weight at 1st, 2nd, 3rd months after surgery between santulli and control groups (Table 5). The percentiles for weight at 6th, 9th months after surgery in the santulli group were significantly higher (Figure 3). The length of incision at 1 years old in the santulli group was shorter than that in the control group.

**Table 5:** Outcome variables during follow-up in both Santulli and control groups.

Parameter	Santulli group (*n* = 33)	Control group (*n* = 43)	*χ* ^2^	*P*
^F^Adhesive ileus	1 (3.03)	5 (15.15)	----	0.225
Length of insion (mm)	130.94 ± 13.88	150.58 ± 22.34	4.703	<0.001
**Percentiles for weight**				
At stoma	43.06 ± 24.88	45.21 ± 26.24	0.364	0.717
1st months after stoma	37.21 ± 25.01	39.35 ± 27.25	0.355	0.724
2nd months after stoma	37.82 ± 23.22	34.58 ± 30.10	0.529	0.598
3rd months after stoma	40.88 ± 22.54	34.63 ± 31.63	1.005	0.318
6th months after stoma	45.03 ± 21.14	29.30 ± 23.18	3.082	0.003
9th months after stoma	50.82 ± 20.37	33.60 ± 20.26	3.660	<0.001
12th months after stoma	48.39 ± 19.11	42.28 ± 19.93	1.357	0.179

F, Fisher's exact test.

## Discussion

In several cases, despite an appropriate procedure to restore intestinal continuity, digestive autonomy might be delayed because of intestinal dysmotility. For patients with intestinal atresia, NEC-related obstruction, or dysfunctional anastomosis in other intestinal congenital diseases, Schafer (11) showed that stimulating the distal segment by feeding before anastomosis demonstrates a positive effect on the intestinal wall, mucosal growth, lumen size, and stoma reversal conditions. Nicolas (12) reported that the Santulli procedure is safe and effective in neonates to allow for a quick recovery after stoma closure, thus supporting the concept that progressive stimulation and irrigation of nutrients and feces into the defunctionalized distal bowel may improve the delay in achieving digestive autonomy in pediatric patients affected by congenital intestinal malformation or NEC. Although it requires an additional procedure compared to direct anastomosis, it prevents episodes of intestinal distension linked to dysmotility, bacterial translocation, or even catheter-related sepsis due to PN dependence. This study compared the clinical efficacy and adverse events between Santulli enterostomy and double-/single-lumen ostomy for various surgical emergencies of newborns that necessitated stoma and evaluated the indications of Santulli procedure.

**Figure 4:**
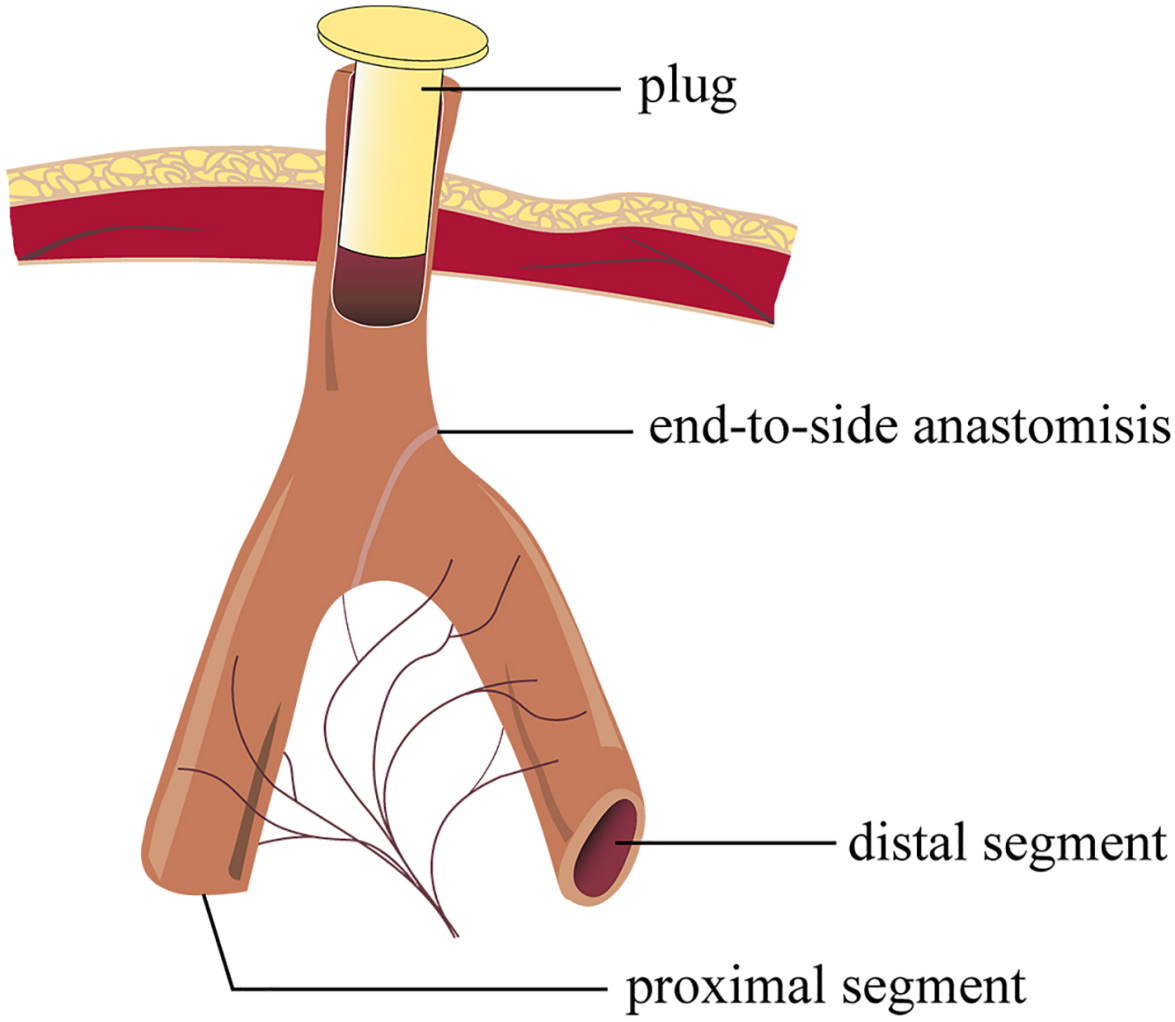
Diagram of enclosing the vent of Santulli stoma.

In Santulli enterostomy, the proximal bowel was brought out as an enterostomy and an end-to-side fashioned anastomosis. Compared with double-/single-lumen ostomy, the Santulli enterostomy procedure is relatively complex; additionally, the duration of enterostomy procedure in the Santulli group is longer when compared with the control group (136.30 vs. 119.00, *P* = 0.108). Nevertheless, closure of the Santulli stoma is a simple procedure that can be carried out without major peritoneal breeching. Briefly, the common blind end-stoma is mobilized away from the skin, subcutaneous tissue, and fascia. Additionally, it is closed just below the fascia level. Enclosing the ostomy (Figure 4) is a useful way to examine the distal bowel movement before closing the Santulli stoma. If the distal bowel movement is well, the secondary surgery needed only involves stitching of the exposed arm of “T” enterostomy, and releasing the intraperitoneal adhesion would be unnecessary. Stitching of one arm of the “T” enterostomy means side anastomosis, and it could be performed by endo-GIA, which is simple to perform. Overall, the stoma closure was simple and the requirement of closure for patients’ tolerance in Santulli stoma was undemanding. Compared to the control group, the operative time of stoma closure is shorter (53.00 vs. 152.47, *P* < 0.001), the bleeding is lesser (4.70 vs. 15.93, *P* < 0.001), the ratio of secondary intestinal adhesions and the ratio of anastomotic fistula are lower in the Santulli group of this cohort. Additionally, Patients were discharged home after a mean time of 7.64 days following stoma closure in the santulli group, that was significantly fewer than in the control group, and the length of incision at 1 years old in the santulli group was shorter than that in the control group, it is also important for the aesthetic requirements of parents. In view of the above, stoma closure of Santulli group could be performed in advance, shortening the interval between enterostomy and closure in the Santulli group (131.21 vs. 216.42, *P* < 0.001) don't increase complication and length of stay after stoma close (Table 3), which is helpful to shorten the total duration of the treatment.

Reports have shown that high output ostomy is a risk factor for malnutrition after neonatal enterostomy. Additionally, postoperative diarrhea and dehydration are the most common causes of unplanned readmission of children with enterostomy (13). Early distal refeeding provides an environment conducive to adaptation of the remnant bowel, which occurs *via* early increases in crypt depth, villus height, and crypt fission as well as permanent increases in the number of crypts and bowel caliber. Compared to the control group, the day of starting oral or nasal feeding in the santulli group was significantly fewer, With a proximal end enterostomy to effectively decompress the proximal bowel and permit an early instillation of nutrients into the distal bowel, the Santulli enterostomy avoids the non-use of the distal bowel, especially the terminal ileum and colon. Therefore, it could restore enterohepatic circulation, preserve the intestinal microbiota, avoid diversion colitis, and then reduce the risk of cholestasis, sodium depletion, and metabolic acidosis, especially in SBS (14, 15). It prevents any risk of bowel overload. Recent support was provided to such “in continuity” enterostomies to improve the bowel function in SBS (16). Better than nutrients, the proximal content also brings biliary and pancreatic secretions that will improve the rate of intestinal absorption (17, 18). Occlusion of the vent of santulli stoma could lengthen the available bowel, It also persist the terminal ileum remains intact, this can reduce the stomal output, increase the absorption of nutrients, improve weight gain, and reduce the requirements of PN usage. In the study, although there were no difference in comparision of the level of enterostomies and the rate of high-sight stoma between the two groups, but the electrolyte disturbances were significantly severer in the control group, and all the nutritional indexes, including Retinol-binding protein, Prealbumin, Vitamin B12, were significantly better in the santulli group (Table 4). The ratio of high output ostomy in the control group was significantly more than that in the Santulli group (10 vs. 2, *P* = 0.042), cases who underwent high output ostomy need a longer duration of PN to maintain their fluid and electrolyte acid-base balance, and improve the nutritional status, so that the time to discharge of the control group was significantly longer than that of the Santulli group (67.60 vs. 46.00, *P* < 0.001), the results show good agreement with literature data. After a median follow-up of 36 (12–72) months, the percentiles for weight at 6th, 9th months in the santulli group were significantly higer than that of the control group (Figure 3), that means the catch-up growth was faster in the santulli group, which is important for infant development. In the 10 cases of the control group with high output ostomy, seven cases were of repeated readmissions for parenteral nutrition because of hydroelectrolyte disturbance and malnutrion; two of these cases were forced to undergo closure of the ostomy ahead of time, three cases could not be discharged and underwent a second surgery after receiving parenteral nutritional supplementation in the hospital for approximately 3 months (one case underwent stoma closure, two cases underwent the Santulli procedure). Taken together, the Santulli stoma is helpful for the absorption of nutrients, improve weight gain and reduce some of the requirements for PN, which is the reason behind why the duration of PN and length of stay were both significantly shorter in the Santulli group, and reduces PN-associated complications (2 vs. 8, *P* = 0.109).

De Jorge (19) reported that massive septic contamination of the abdominal cavity increases the risk of leakage from any intestinal suture. In fact, there was no leakage in Santulli enterostomy patients of this cohort. Therefore, Santulli enterostomy is determined to be safe for acute NEC. Several surgeons did not consider Santulli enterostomy during the acute phase of NEC, intestinal perforation, and meconium ileus, because it results in the same morbidity as primary anastomosis and requires additional operating time (20). In this study, the operative time of Santulli enterostomy is longer than that in the control group; however, the operative time of stoma closure in Santulli stoma is significantly lesser. On the other hand, adhesive intestinal obstruction and intestinal stenosis are common in patients suffering from intestinal spontaneous perforation, NEC, and meconium ileus. Therefore, surgeons should ascertain whether the bowel movement is normal. By enclosing the vent of santulli stoma, it is easy to figure out the defecation situation. Once there was no secondary movement complication, the closure was simple and safe. The operative time, rate of complications, and LOS were lesser than that of the control group.

Proximal bowel dilatation is common in intestinal atresia requiring additional bowel resection or a tapering procedure before entero-anastomosis (21, 22). Even if an end ostomy was performed, the discrepancy of the diameter in the two sides in the second anastomosis will still be significant, which might lead to a funnel-shaped anastomosis, which would increase the risk of anastomotic fistula, prone to anastomotic obstruction, and delay digestive autonomy, even to reoperation, et al. one increase the difficulty of anastomosis during the second surgery. In the situation wherein the proximal bowel is sufficiently dilated and bowel resection is not possible, exteriorization with later attempted closure is advisable (23). However, if the dilated intestinal area is near the duodenojejunal junction, a high output would be faced inevitably; this issue can be resolved by the Santulli procedure perfectly. The maximum diameter ratios in the proximal end to the distal end in this cohort was 12 times (Table 2); the diameter ratio is similar in the two groups before stoma (9.77 vs. 9.45, *P* = 0.638); however, the diameter ratio in the Santulli group was smaller (2.48 vs. 5.91, *P* < 0.001), the difference was statistically significant. The Santulli procedure promotes the circulation of the intestinal contents to the distal end, retains the function of the distal intestinal mucosa, reduces the waste atrophy of the distal intestinal tube, and promotes the development of the distal intestinal tract. At the same time, the proximal bowel tapered while it was unobstructed, such that the Santulli procedure can diminish the discrepancy between the proximal and distal segments (Figure 1), and then decrease the difficulty of anastomosis and the risk of anastomotic fistula, bowel obstruction, and SBS. Therefore, the Santulli procedure is a better-suited procedure for intestinal atresia.

It is well-known that for cases whose intestinal lesion is less than 10 cm from the ileocecal junction, primary anastomosis means a high risk of fistula because of the ileocecal valve. Therefore, several surgeons prefer to perform ileocecal junction resection and ileocolostomy. In fact, the ileocecal junction and the terminal ileum are important for intestinal absorption for infants. A total of eight cases with lesions near the ileocecal junction underwent the Santulli procedure in this study. The ileocecal junction of these patients was preserved and no anastomosis fistula occurred. It shows that the Santulli procedure is helpful to preserve the ileocecal junction for the case whose lesion is near the ileocecal junction.

For patients whose situation of ganglion cell in distal bowel is uncertain, multiple-spots biopsy is a common choice; however, it increases the risk of bowel stenosis and leaks because the bowel of neonates is thin, and various factors affect the accuracy of biopsy. In the Santulli group, ostomy closure could be accomplished quickly without contrast enema for the case whose bowel movement was normal after enclosing the vent, what commonly means normal ganglion cell. For cases who suffer from intestinal obstruction after enclosing the vent, the reason, including abnormal ganglion cell, intestinal stenosis or kinks, should be inspected. The Santulli procedure is helpful to observe the movement of the distal bowel for the cases with uncertain ganglion cells.

This study has a few limitations. First, the study was carried out at a single institution. The study was of a retrospective design and the cohort of the study was small. Larger randomized comparative trials will be needed to demonstrate the superiority for the approach for the treatment of surgical intestinal diseases in newborns.

In conclusion, Santulli enterostomy is feasible and safe. It is associated with lesser complications, better postoperative nutrition, shorter duration of intravenous nutrition, rapid recovery, and lower costs than other procedures. It has distinct superiority for surgical intestinal diseases in newborns, especially in the case of patients with NEC, meconium ileus, intestinal atresia, lesion near to ileocecal junction or uncertain ganglion cell in the distal bowel.

## Data Availability

The original contributions presented in the study are included in the article/Supplementary Material, further inquiries can be directed to the corresponding author.
